# Atypical Proximal Gallstone Ileus Near the Ligament of Treitz Managed With Staged Surgical Intervention: A Case Report

**DOI:** 10.7759/cureus.106117

**Published:** 2026-03-30

**Authors:** Joseph V Marzano, Michael Sauder, Frances McCarron, Lillianne Stanitsas

**Affiliations:** 1 Anesthesia, Lake Erie College of Osteopathic Medicine, Erie, USA; 2 General Surgery Services, Mercy Health - St. Elizabeth Youngstown Hospital, Youngstown, USA

**Keywords:** biliary-enteric fistula, bouveret's syndrome, gallstone ileus, ligament of treitz, proximal obstruction, small-bowel obstruction, staged surgical correction

## Abstract

Gallstone ileus is a rare mechanical bowel obstruction caused by the migration of a gallstone through a biliary-enteric fistula, most commonly impacting at the ileocecal valve. Proximal obstruction is uncommon and is typically classified as Bouveret syndrome when involving the stomach or proximal duodenum. Obstruction near the ligament of Treitz represents an exceedingly rare variant.

A 76-year-old female presented with nausea, vomiting, and abdominal distention. Computed tomography demonstrated gastric and proximal small bowel dilation secondary to an obstructing gallstone in the distal duodenum. After anticoagulation reversal, exploratory laparotomy was performed. The stone was manually advanced into the jejunum, followed by segmental resection and primary anastomosis. The gallstone measured 6 cm. The gallbladder and fistula were not addressed initially. The patient recovered well and was discharged on postoperative day four. She later underwent an elective robotic cholecystectomy with fistula takedown without complication.

This case describes an atypical proximal gallstone ileus located near the ligament of Treitz, distinct from both classic gallstone ileus and Bouveret syndrome. A staged surgical approach allowed safe relief of obstruction followed by definitive biliary management.

This case highlights the importance of individualized surgical planning and supports staged management as a safe and effective strategy for rare proximal gallstone ileus.

## Introduction

Gallstone ileus is a mechanical obstruction of the gastrointestinal tract, usually due to the passage of a gallstone through a biliary-enteric fistula, ultimately becoming lodged in the digestive tract. This classically occurs as a sequela of chronic cholecystitis, in which pericholecystic inflammation leads to the formation of adhesions between the gallbladder and stomach or bowel. Subsequently, pressure necrosis from a large gallstone may form a fistula between the biliary system and the enteric tract [1].

Although more than 25% of women older than 60 are estimated to have gallstones, gallstone ileus is a rare condition, occurring in only 0.3-0.5% of patients with gallstones [2]. The vast majority of gallstone ileus cases occur with obstruction at the ileocecal valve; however, there is limited literature on other sites of obstruction [1].

Bouveret syndrome is a rare variant of gallstone ileus in which a gallstone becomes impacted in the stomach or proximal duodenum [3]. This condition most commonly affects elderly females. A wide variety of presenting symptoms have been reported, including acute pancreatitis, upper gastrointestinal bleeding, duodenal perforation, and esophageal rupture [3]. In general, larger stones can result in more proximal obstruction. Accordingly, stones associated with Bouveret syndrome are typically greater than 2.5 cm in size [4].

The following case describes a 76-year-old female who presented with an obstructing gallstone lodged in the distal duodenum, just proximal to the ligament of Treitz. This case is unique due to the location of the obstruction. While the obstructing stone is more proximal than a typical gallstone ileus, the point of obstruction is also too distal to be considered true Bouveret syndrome. Although a case report of a similar obstruction was published in March 2025 [5], our case differs in terms of surgical management. Thus, reporting this case will add to the literature on alternative approaches to surgical management of proximal gallstone obstruction.

## Case presentation

A 76-year-old female presented to the emergency department for evaluation of three days of nausea, vomiting, and abdominal distention. She denied prior episodes of similar symptoms or a history of postprandial abdominal pain. Her medical history was significant for hypertension, hyperlipidemia, and atrial fibrillation. Surgical history was notable for hysterectomy. Home medications included apixaban, metoprolol, lisinopril, and empagliflozin. On physical examination, the patient had significant abdominal distention with minimal abdominal pain. Her initial laboratory work did not demonstrate a significant elevation in liver function tests or serum bilirubin.

**Figure 1:**
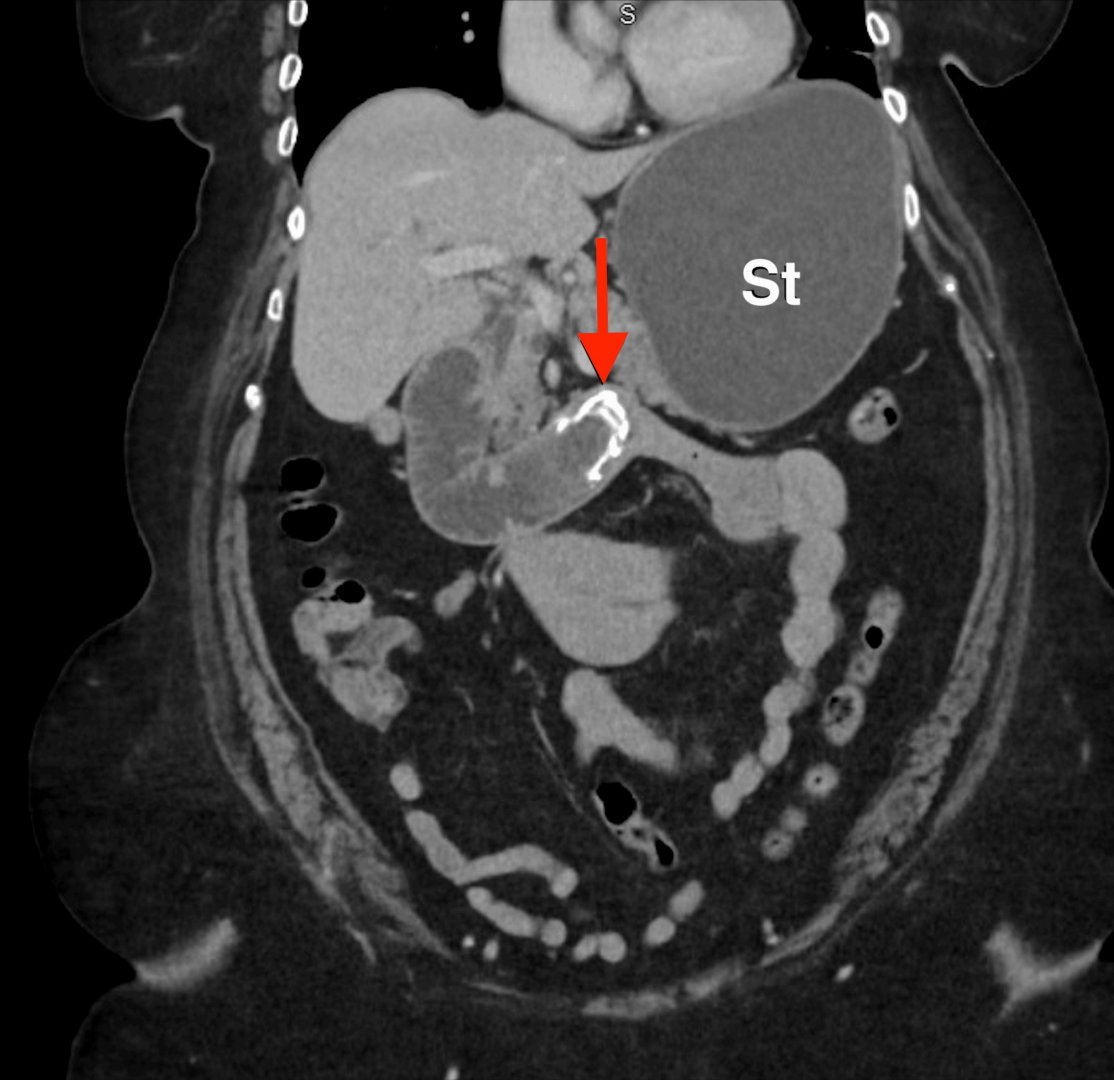
Coronal contrast-enhanced CT of the abdomen demonstrating a hyperdense calculus within the duodenal lumen (arrow), consistent with an ectopic gallstone causing proximal obstruction with stomach (St) distention.

**Figure 2:**
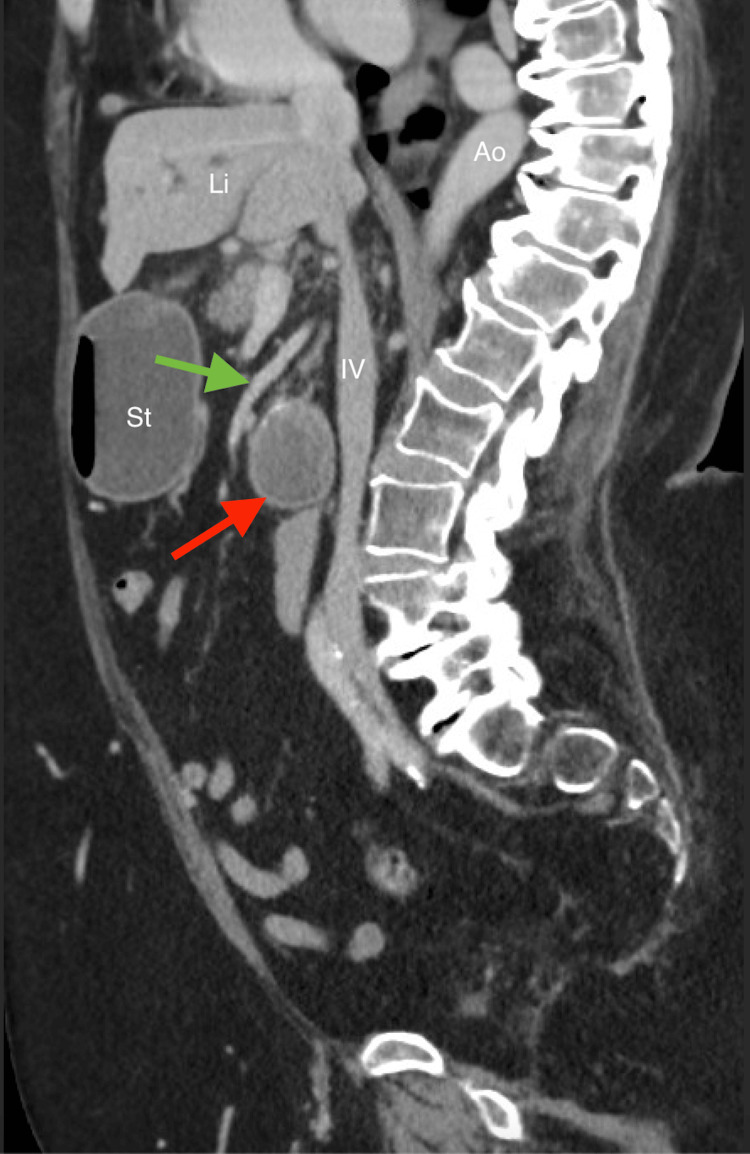
Contrast-enhanced sagittal CT of the abdomen demonstrates a large, rounded hyperdense calculus within the duodenal lumen (red arrow), consistent with an ectopic gallstone, resulting in adjacent stomach (St) is distention. The superior mesenteric artery (SMA; green arrow) is seen coursing anterior to the duodenum, with the aorta (Ao), the inferior vena cava (IV) and liver (Li) labeled for anatomic orientation.

**Figure 3:**
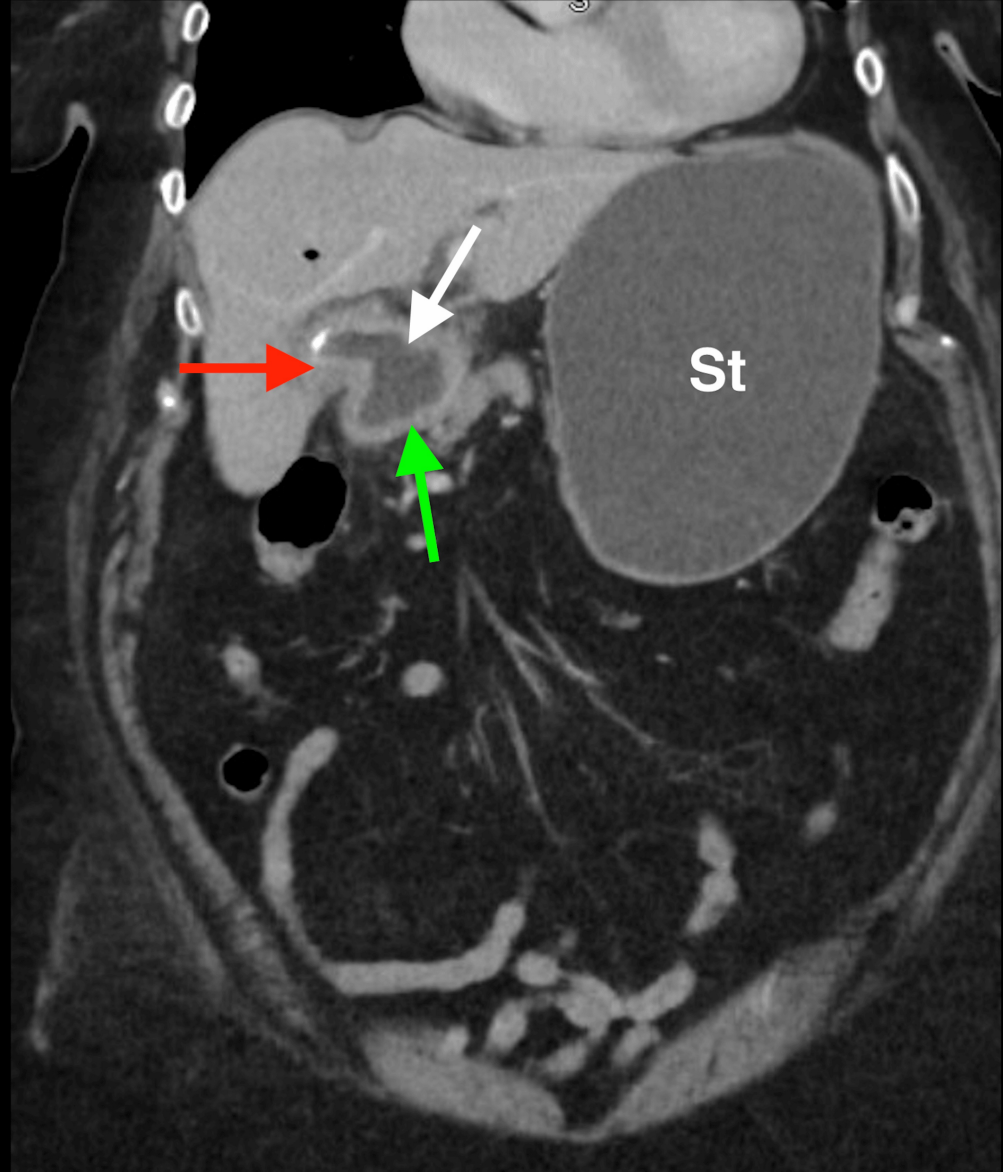
Coronal contrast-enhanced CT of the abdomen demonstrating a biliary-enteric fistula. The red arrow highlights the gallbladder, the green arrow indicates the adjacent duodenum, and the white arrow demonstrates the fistulous communication between the gallbladder and duodenal lumen, consistent with a cholecystoduodenal fistula. The stomach (St) is labeled for anatomical orientation.

CT imaging of the abdomen and pelvis demonstrated gastric and proximal bowel dilation due to an obstructing stone in the distal duodenum, posterior to the superior mesenteric artery (Figures 1, 2). Coronal view revealed a biliary-enteric communication between the gallbladder and the adjacent duodenum, consistent with a cholecystoduodenal fistula (Figure 3). These findings supported biliary-enteric communication in the setting of gallstone ileus. 

The patient was given broad-spectrum antibiotics as well as prothrombin complex concentrate to reverse her anticoagulation. A nasogastric tube was inserted for gastric decompression. She was subsequently taken to the operating room for exploratory laparotomy. Intraoperatively, a large, firm mass was palpated within the lumen of the small bowel just proximal to the ligament of Treitz.

**Figure 4:**
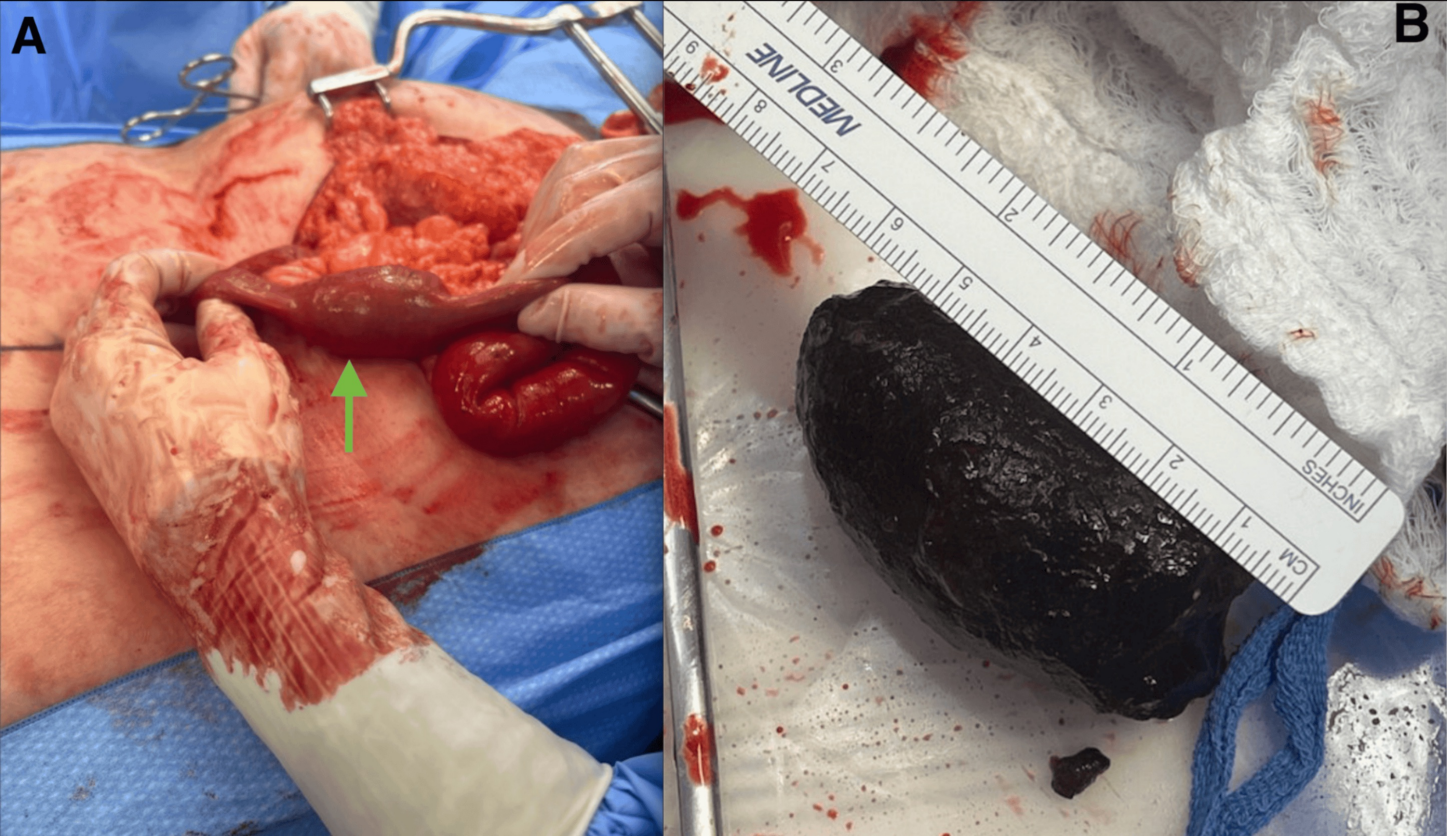
Intra-operative and specimen photographs demonstrating obstructing gallstone managed with segmental jejunal resection. (A) Intra-operative image demonstrating a large intra-luminal gallstone (arrow) after it was milked into the jejunum. (B) The gross specimen of the surgically removed gallstone measuring 6 cm, which was the cause of the intestinal obstruction.

Using gentle manual pressure, the mass was propelled through the bowel lumen into the jejunum (Figure 4A). A short segment of jejunum containing the mass was then resected using a surgical stapler. The resected specimen was opened, revealing a 6.1 x 3.1 x 3.0 cm gallstone (Figure 4B). A stapled anti-peristaltic side-to-side anastomosis was created between the remaining bowel segments, and the mesenteric defect was closed with suture. This anastomotic approach was chosen so the segment would lie anatomically in the left upper quadrant without tension or twisting. Evidence of extensive chronic pericholecystic inflammation with an adherent duodenum was present, and the gallbladder was left in situ. The small bowel was examined in its entirety without evidence of additional pathology. The abdomen was then closed. The pathological report of the gallstone did not disclose the composition of the stone. 

The patient was seen in follow-up by a hepatobiliary surgeon, who ultimately performed robotic-assisted laparoscopic cholecystectomy, takedown of the cholecystoduodenal fistula, and modified Graham patch repair of the duodenum approximately three months after the previously described laparotomy. Following this procedure, the patient was discharged home in good condition on postoperative day 2. She has since been seen in follow-up and is doing well, without current abdominal complaints.

## Discussion

**Figure 5:**
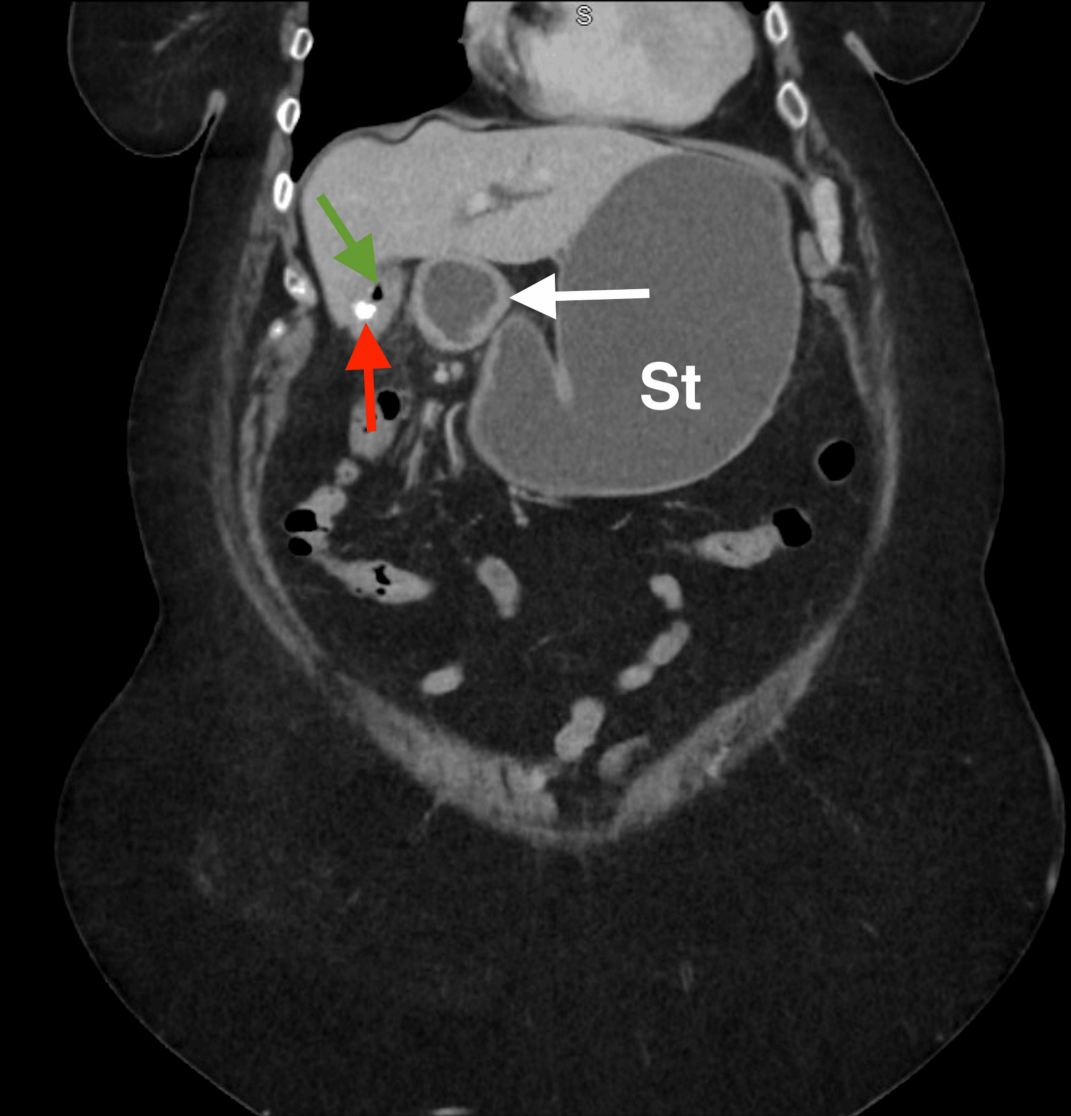
Coronal contrast-enhanced CT of the abdomen demonstrating Rigler’s triad in gallstone ileus. The green arrow indicates pneumobilia with air within the gallbladder/biliary system, the red arrow highlights gallstones within the gallbladder. The white arrow demonstrates the distended duodenal lumen, and the stomach (St) is markedly distended, consistent with distal duodenal obstruction.

Due to the uncommon nature of this case, the currently available literature lacks a clearly defined algorithm for surgical management, which is why this case is important in guiding the management of future cases. CT imaging demonstrating Rigler’s triad (bowel obstruction, ectopic gallstone, and pneumobilia) remains the most sensitive diagnostic modality for gallstone ileus (Figure 5) [6]. The main therapeutic goal in managing gallstone ileus is to remove the obstructing gallstone. While endoscopic approaches have been described for cases of Bouveret syndrome, the obstructing gallstone in the present case was lodged too distally and was too large for endoscopic retrieval. Overall, endoscopic intervention has been reported to have a low success rate (<10%) in the management of obstructing gallstones [7]. While endoscopic lithotripsy has been described, this was not pursued in this case due to the distal location of the obstructing stone as well as the risk of migration of large stone fragments, with the potential to cause further distal obstruction [8]. 

Laparoscopic approaches to the management of both gallstone ileus and Bouveret syndrome have been described [7]. However, in the presented case, an open procedure was the safest approach due to the large size of the impacted gallstone as well as its retroperitoneal location with proximity to major vasculature. Additionally, the patient had recently taken her oral anticoagulant.

The timing and management of the biliary-enteric fistula are also debated in the literature. Options include stone extraction without a definitive biliary procedure, stone extraction followed by cholecystectomy and fistula takedown, and a one-stage procedure combining stone extraction with cholecystectomy and fistula takedown. While the reported mortality rate for stone extraction alone is 12%, definitive one-stage procedures have been associated with a 20% mortality rate [9-11]. This high associated mortality is likely influenced by the frail and deconditioned demographic most commonly affected by gallstone ileus.

In our case, the patient’s definitive biliary procedure required extensive dissection, which was successfully performed by a hepatobiliary specialist through a minimally invasive approach once the patient was better optimized. The patient did not have evidence of acute cholecystitis; however, she did have radiologic evidence of residual stones within the gallbladder (Figure 5). Both of these factors played a role in the decision-making process. It should be noted that no further analysis of the composition of the stone was performed after resection, which is a potential limitation of the study.

## Conclusions

This case highlights gallstone ileus with an atypical site of obstruction near the ligament of Treitz, an uncommon presentation that exists in a diagnostic and therapeutic space between classic gallstone ileus and Bouveret syndrome. Because proximally obstructing gallstones are rare and may produce variable clinical manifestations, optimal management often requires individualized decision-making rather than adherence to a single standardized approach. Given the significant morbidity and mortality associated with this condition, early recognition and careful operative planning are essential. This case underscores the importance of maintaining a broad differential diagnosis in elderly patients presenting with gastric outlet or proximal small bowel obstruction and illustrates how a staged surgical strategy can safely address both the obstructing calculus and the underlying biliary-enteric fistula.
